# Comprehensive understanding of the effects of metallic cations on enzymatic hydrolysis of humic acid-pretreated waste wheat straw

**DOI:** 10.1186/s13068-021-01874-5

**Published:** 2021-01-19

**Authors:** Wei Tang, Xinxing Wu, Caoxing Huang, Zhe Ling, Chenhuan Lai, Qiang Yong

**Affiliations:** 1 Jiangsu Co-Innovation Center of Efficient Processing and Utilization of Forest Resources, College of Chemical Engineering, Nanjing Forestry University, Nanjing, 210037 People’s Republic of China; 2 Key Laboratory of Forestry Genetics and Biotechnology (Nanjing Forestry University), Ministry of Education, Nanjing, 210037 People’s Republic of China

**Keywords:** Humic acids, Metallic cations, Cellulase, Enzymatic hydrolysis

## Abstract

**Background:**

Humic acids (HA) have been used in biorefinery process due to its surfactant properties as an aid to the pretreatment of lignocellulose, with results indicating a positive effect on delignification. However, the HA remaining on the surface of the pretreated lignocellulose has also been shown to provide a negative effect on ensuing enzymatic digestibility. Hence, a strategy of complexing metallic cations with HA prior to enzymatic hydrolysis was proposed and demonstrated in this work in an effort to provide a means of HA mitigation that does not involve significant water consumption via extensive washing.

**Results:**

Results showed that the enzymatic hydrolysis efficiency of waste wheat straw decreased from 81.9% to 66.1% when it was pretreated by 10 g/L HA, attributed to the inhibition ability of the residual HA on enzyme activity of cellulase with a debasement of 36.3%. Interestingly, enzymatic hydrolysis efficiency could be increased from 66.1% to 77.3% when 10 mM Fe^3+^ was introduced to the system and allowed to associate with HA during saccharification.

**Conclusions:**

The addition of high-priced metallic cations (Fe^3+^) has successfully alleviated the effect of HA on cellulase activity. It is our hope in demonstrating the complexation affinity between metallic cations and HA, future researchers and biorefinery developers will evaluate this strategy as a unit operation that could allow economic biorefining of WWS to produce valuable biochemicals, biofuels, and biomaterials.

## Background

The efficient development and utilization of bioenergy will allow humanity to better address the energy and ecological problems it currently faces [1, 2]. Bioenergy has a wide range of sources, including forestry resources, agricultural resources, industrial waste, municipal solid waste, and livestock manure [3, 4]. As a part of agricultural resources, wheat straw has attracted significant interest for its utility as a bioenergy feedstock due to its worldwide abundance [5]. One practice used in China is to manufacture paper from wheat straw. However, some residues are abandoned during this process. These abandoned materials consist of wheat leaves, ears, straw scraps, and free ash, all of which are screened out and collective, known as waste wheat straw (WWS). WWS is chemically composed of cellulose (24–28%), hemicellulose (20–22%), lignin (18–22%), and ash (28–30%) [6]. The abundance of carbohydrates in the WWS renders it a promising candidate for biomass-based fuels or biochemicals. Major difficulties in the bioconversion scheme for WWS include its intrinsic heterogeneity, as well as its recalcitrance toward cellulolytic enzymatic hydrolysis [7]. To address the recalcitrance, a pretreatment operation is necessary to eliminate the physical barriers preventing WWS from further disassembly. In previous studies, the compact structure of WWS has been conquered through application of autohydrolysis pretreatment, which is an economical and ecofriendly pretreatment technology [8, 9]. However, a large amount of ash present in WWS presented its own set of challenges for autohydrolysis due to a hydrolyzate-buffering effect [10, 11].

A relatively simple method for dealing with this problematic effect provided by ash is prewashing, which was previously investigated in earlier works [6]. 500 mL/g WWS water consumption can increase the enzymatic hydrolysis efficiency by 30.11%. While the ash is regarded as a problem for utilization of WWS, one key finding from a recent work was that the organic portion of the ash in WWS actually provides a positive effect on the pretreatment by assisting delignification [12]. These organic components in the ash fraction are mainly humic acids (HA). The basic structure of the HA is an aromatic ring and an alicyclic ring, functional groups (mostly hydroxyl and carboxyl group) is attached to the ring [13]. HA is a complex mixture of high weight hydrophobic and hydrophilic molecules, which function as natural surfactants due to their amphiphilic properties [13, 14]. Research on surfactants in the field of biorefinery mainly focuses on two points. Firstly, surfactants can enhance substrate hydrophilicity by reducing the surface tension between two liquid phases during the pretreatment [15, 16]. Secondly, surfactants have also been shown to enhance cellulolytic enzymatic digestion of lignocellulosic substrates by reducing non-specific adsorption of lignin on the surface of lignocellulose [17, 18]. Currently, the research on surfactants in the field of biorefinery is more inclined to synthetic surfactants, i.e., PEG, SDS, Tween rather than natural surfactants. The utility of naturally derived surfactants, like HA, has actually garnered significant industrial interest over the past decade due to its non-toxic, ecologically safe, and high surface activity [19]. It is this value-added property of an otherwise problematic element of WWS that we believe will assist in the industrial realization of a WWS-based biorefinery process.

The addition of neat HA into an autohydrolysis medium has been performed using prewashed WWS in our previous work, and we found that the HA has a positive effect on delignification during the autohydrolysis of WWS [20]. This is mainly due to the fact that HA acted as a co-solvent to solubilize the hydrophobic polymers. However, it was also found that pretreated WWS with HA was unable to achieve sufficient great conversion of cellulose due to the presence of residual HA adsorbed onto the surfaces of the WWS. It has been reported that HA can interact with proteases, lead to modification of the protein structure and character, consequently, change protein biological activity [21, 22]. Therefore, the HA remaining on the surface of WWS requires a lot of water to clean to enhance the downstream enzymatic hydrolysis efficiency. Considering the economic cost of biorefinery, it is necessary to find a simple additive to eliminate the adverse effects of HA on enzymatic hydrolysis. The HA can interact with a variety of solutes due to its complex functional groups, including hydrophobic interaction, chelation or complexation [23]. Preeminent among the interactions of HA with solutes are metallic cations [24]. The metallic cations bonded with HA through cation exchange and electrostatic force adsorption to form complexes or chelates [25]. Therefore, whether the negative effect of HA on enzymatic hydrolysis can be eliminated by adding metallic cations during the enzymatic hydrolysis is worth further investigation.

In this work, the negative effect of HA on cellulase activity has been investigated. Next, different metallic cations (K^+^, Na^+^, Ca^2+^, Fe^2+^, Fe^3+^, Al^3+^) were introduced into the HA colloid solution to determine its effect on the enzyme activity. Furthermore, the zeta potential and dynamic viscosity of the suspension formed by metallic cations and HA were measured to prove the inner interactions. At last, the best-performing cations were added into the enzymatic hydrolysis of HA-pretreated WWS to overcome the effects of HA on cellulase. This work is intended to further knowledge into the complicated topic of utilizing humic acids in a way the benefits the overall prospects of a biorefining process based on WWS.

## Results and discussion

### Compositional analysis of WWS and pretreated AWWS

It is first important to understand how chemical compositions of our test materials vary as a function of the processes they are subjected to. The prewashed WWS was analyzed for chemical composition, and the results are included in Table 1. Specifically, this material was composed of (on a dry basis) 36.0% glucan, 26.1% xylan, 23.3% lignin (20.1% acid-insoluble lignin and 3.2% acid-soluble lignin) and 9.1% ash. Next, washed and unwashed autohydrolyzed WWS (AWWS) in the presence of 10 g/L HA was analyzed. The washed AWWS was found to comprise 49.5% glucan, 11.6% xylan, and 30.1% lignin (29.1% acid-insoluble lignin and 1.0% acid-soluble lignin), and the unwashed AWWS was found to comprise 44.2% glucan, 10.1% xylan, and 28.7% lignin (27.7% acid-insoluble lignin and 1.0% acid-soluble lignin). Meanwhile, the quantity of residual HA in unwashed AWWS was measured to be 0.05 g/g substrate, while the HA was not detected in the washed AWWS. It displayed that the difference between washed and unwashed AWWS was the fraction of HA. Whether this residual HA can be beneficial to downstream cellulase hydrolysis of AWWS like other active agents, needs to be verified by saccharification of washed and unwashed AWWS.

**Table 1:** The composition of raw WWS and AWWS

Substrate	Glucan (%)	Xylan (%)	Acid-soluble lignin (%)	Acid-insoluble lignin (%)	Ash (%)	HA (%)
Raw WWS^a^	26.2 ± 0.2	21.7 ± 0.1	3.0 ± 0.3	17.4 ± 0.1	29.5 ± 0.1	\
Prewashed WWS	36.0 ± 0.1	26.1 ± 0.2	3.2 ± 0.4	20.1 ± 0.5	9.1 ± 0.2	\
Unwashed AWWS^b^	44.2 ± 0.4	10.9 ± 0.5	1.0 ± 0.2	27.7 ± 0.2	9.2 ± 0.5	5.0 ± 0.4
Washed AWWS	49.5 ± 0.2	11.6 ± 0.1	1.0 ± 0.5	29.1 ± 0.2	1.4 ± 0.4	0.0 ± 0.0

^a^WWS was waste wheat straw; ^b^AWWS was autohydrolysis waste wheat straw with 10 g/L HA

### The effects of residual HA on enzymatic hydrolysis of AWWS

**Fig. 1:**
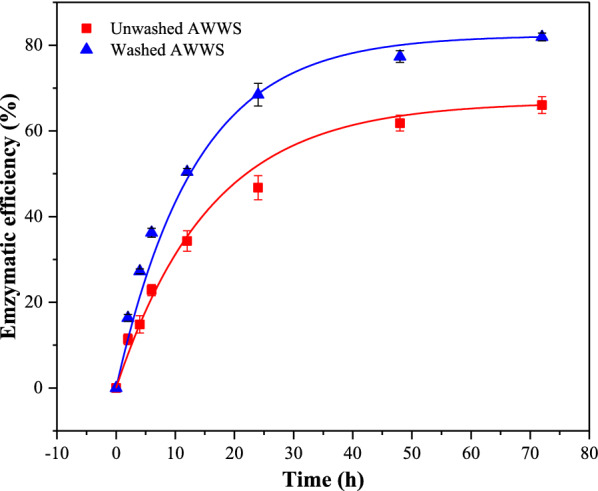
The enzymatic hydrolysis of washed and unwashed AWWS

Enzymatic hydrolysis is a key step in biorefinery operations, which requires a consortium of different enzymes that can hydrolyze cellulose and hemicellulose into fermentable monosaccharides [26]. In the context of this work, enzymatic hydrolysis was performed on unwashed and washed AWWS to estimate the effect of residual HA on enzymatic hydrolysis. Results are shown in Fig. 1. The enzymatic hydrolysis efficiency of unwashed AWWS was 66.1%, while that of washed AWWS was 81.9%. The results showed that the act of thoroughly washing the substrate had a prominently positive effect on the cellulose conversion of the pretreated samples. This is most likely due to the remaining HA attached to the surface on the unwashed AWWS affecting the cellulase’s activity. The operation of washing can successfully remove the residual HA, and then increase the conversion rate of cellulose. Washing is considered to be the easiest way to remove the non-sugar compounds (i.e., furan derivatives, carboxylic acids, phenolic compounds) that have a negative effect on downstream enzymatic hydrolysis and fermentation process after the pretreatment of lignocellulose [27, 28]. However, the washing operation consumes a lot of water, causing waste of resources and environmental problems [4, 29]. In order to address the influence of residual HA on enzymatic hydrolysis efficiency, HA’s effects on cellulase activity were firstly investigated.

### The effects of HA on cellulase activity and protein adsorption

**Fig. 2:**
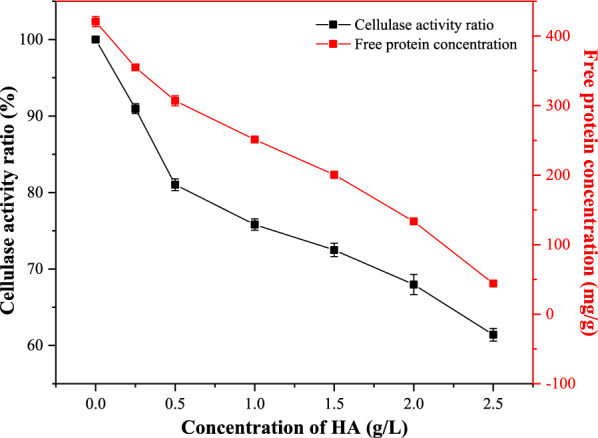
The cellulase activity ratio and adsorbed protein amount of the HA and cellulase suspension

**Fig. 3:**
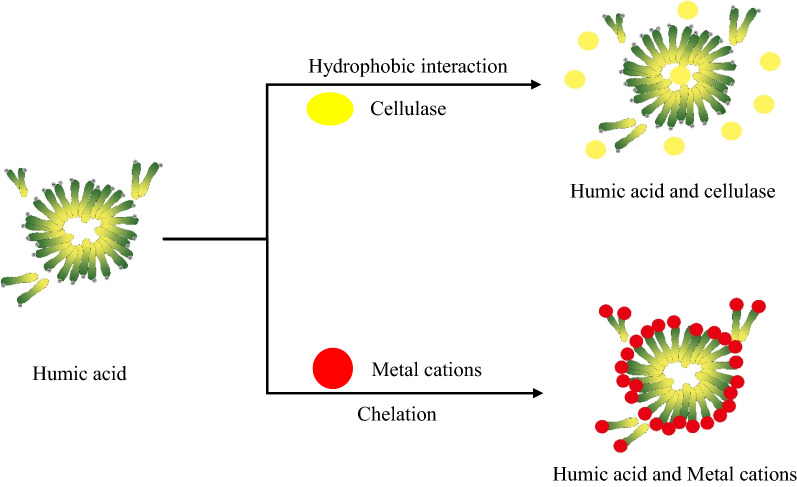
Diagram of the interaction between HA with cellulase and metallic cations

As reported in the literature, filter paper enzyme activity (FPA) is measured as a proxy for the total enzyme activity of cellulase [30]. Given that we sought to learn how HA affects enzymatic activity, experiments were conducted which evaluate FPA of cellulase with and without exposure to HA. As shown in Fig. 2, the FPA decreased from 100% (original enzymatic activity, 225 FPU/g) to 63.7% with HA present at the concentration of 2.5 g/L. This could only be due to the interaction between HA and cellulase affecting its activity. It has been reported that the mechanism by which HA reduced cellulase activity involved the HA either adsorbing domains of the protein or by forming HA–protein complexes [22]. To evaluate the mechanism, the quantity of enzyme protein adsorbed by HA was measured (Fig. 2). Our findings revealed a negative relationship between the quantity of free enzyme protein and the concentration of HA in the system, which indicated that the amount of enzyme protein adsorbed enhanced as the concentration of HA increased. Specifically, we calculated that 376.7 mg of enzyme proteins were adsorbed by HA at a concentration of 2.5 g/L. Previous research has reported that hydrophobic interactions govern the tendency for HA to adsorb to negatively charged proteins (such as insulin, ribonuclease), causing the protein to become encapsulated and lose its functionality [31, 32]. It has also been reported that the interaction between HA and oppositely charged polyelectrolytes has largely been directed to binding by complexation and covalent bonding [33, 34]. However, because cellulase and HA are both polyanions negative charge, it is improbably that polyelectrolyte complexation is playing a role in this system. Instead, it appears that HA is reducing cellulase activity through the aforementioned hydrophobic interactions between them. The probable schematic diagram of the interaction between the HA and cellulase is shown in Fig. 3.

### Complexation of humic acid with metallic cations

From an industrial point of view, the washing step during biorefinery leads to a large consumption of water. This volume will inevitably increase biorefinery processing costs, which may lead to disinterest process industrialization of a WWS-based process. In order to evaluate one possible strategy for mitigating the unfavorable effects of HA on enzymatic hydrolysis, metallic cations were applied to the system in an attempt to complex residual HA present in AWWS, as shown in Fig. 3. Ideally, the complexation will reduce HA’s impact on downstream unit operations (enzymatic hydrolysis). This strategy has been reported previously in the literature [33, 35]. Complexation of different metallic cations (K^+^, Na^+^, Ca^2+^, Fe^2+^, Fe^3+^, Al^3+^) with HA colloidal solutions was evaluated by measuring both zeta potential and dynamic viscosity after complexation. This information was then used to determine which cations are superior for mitigating loss in cellulase activity for more efficient hydrolysis of AWWS.

#### Zeta potential and dynamic viscosity

It has been reported that HA can act as a molecular ligand, providing a coordination bond for complexes with metallic cations [36, 37]. This interaction is usually driven by electrostatic forces [35]. In this work, the zeta potential of the suspension containing HA and various cations was determined to verify electrostatic adsorption. The results can be seen in Table 2. First, we recorded the zeta potential of a HA-only suspension at −26.8 mV. However, when the cations were added, the negative potential was gradually reduced. For some cations, the value reached positivity at the maximum tested cation loading (Fe^3+^ and Al^3+^, 4.8 mV and 3.6 mV, respectively). The higher valence cations likely have greater electrostatic potential that allows for more coordination with HA [22, 37]. Therefore, the higher valence of the metallic cations under the same concentration and the more HA was complexed to it. It has also been reported that HA can be electrostatically adsorbed by metallic cations, causing these complexes to precipitate [38]. This complexation should reduce interactions between HA and cellulase protein.

**Table 2:** The remainder of cations and zeta potential in metallic cations and HA suspensions

Cations	Concentration (mM)	Remainder of cations (mM)	Zeta potential (mV)
None	\	\	−26.8 ± 0.1
K^+^	1	ND	−20.18 ± 0.2
	5	1.5 ± 0.1	−18.3 ± 0.4
	10	4.8 ± 0.2	−16.9 ± 0.2
Na^+^	1	ND	−19.5 ± 0.1
	5	2.7 ± 0.4	−18.4 ± 0.2
	10	6.3 ± 0.2	−17.6 ± 0.2
Ca^2+^	1	ND	−18.4 ± 0.4
	5	0.1 ± 0.2	−11.3 ± 0.6
	10	0.7 ± 0.0	−6.9 ± 0.1
Fe^2+^	1	ND	−14.3 ± 0.1
	5	ND	−4.6 ± 0.5
	10	0.3 ± 0.1	1.3 ± 0.4
Fe^3+^	1	ND	−12.5 ± 0.3
	5	ND	−2.2 ± 0.2
	10	0.1 ± 0.0	4.8 ± 0.2
Al^3+^	1	ND	−14.3 ± 0.4
	5	ND	−4.9 ± 0.5
	10	0.2 ± 0.1	3.6 ± 0.1

**Fig. 4:**
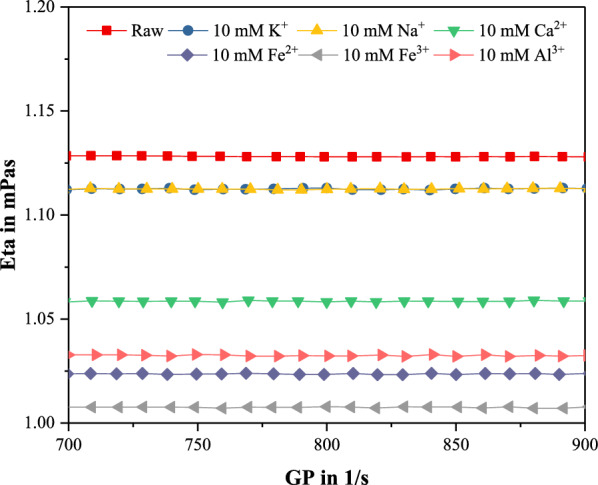
The dynamic viscosity of the suspensions of HA and metallic cations

Dynamic viscosity is another metric that can be used to evaluate the extent of complexation between HA and the tested cations [39]. In order to prove the complexation between HA and metallic cations, the dynamic viscosity of mixed suspension was also measured as reported in the literature [40]. As shown in Fig. 4, the dynamic viscosity of raw HA-only solutions was 1.13 mPa/s. However, the addition of metallic cations reduced the viscosity of the HA solution to different degrees (1.11 mPa/s K^+^, 1.11 mPa/s Na^+^, 1.06 mPa/s Ca^2+^, 1.02 mPa/s Fe^2+^, 1.01 mPa/s Fe^3+^ and 1.03 mPa/s Al^3+^). This is similar to the result of potential, which may be related to the valence and activity of cations. Of the tested cations, Fe^3+^ lowered the viscosity to the greatest extent (1.01 mPa/s, similar to the value of deionized water). According to Ghosh and Schnitzer [41], humic molecules became more rarefied as metal coordination occurs, which they suggested was due to the bridging of neighboring segments of the macromolecules. Therefore, the decrease of the dynamic viscosity in HA suspension was due to lower solute content. Because of the violently complexing ability of Fe^3+^, HA in the solution was completely precipitated, which may be detrimental to the interaction of HA and cellulase.

#### Quantity of metallic cations complexed by HA

To calculate the extent of metallic cation complexation, the concentration of the remainder of metallic cations was detected under the premise of excessive metallic cations concentration (10 mM). It can be seen that the concentration of the residual metallic cations including K^+^, Na^+^, Ca^2+^, Fe^2+^, Fe^3+^, and Al^3+^ all decreased into 4.8 mM, 6.3 mM, 0.7 mM, 0.3 mM, 0.1 mM, and 0.2 mM, respectively (Table 2). These results again showed that the greatest extent of coordination involved cations with higher valences. Trivalent iron was the most adsorbed cation amongst all those tested, with the amount complexed being about 4.0 mM Fe^3+^/g-HA. It has been reported that HA is negatively charged and exists as micelles in the aqueous phase, which can form complexes with protons and metallic cations [22, 42]. According to the Hofmeister series, there is an affinity order for the adsorption of cations and colloidal solutions (Na^+^  < K^+^  < Ca^2+^  < Cu^2+^  < Al^3+^  < Fe^3+^) [43, 44]. This series is in agreement with our observed patterns, including trivalent iron exhibiting the greatest extent of coordination. It has also been reported that the complexing capacity of HA for Al^3+^ and Fe^3+^ was 0.7–3.5 mM/g-HA [45]. All of the previously discussed results and citations were in agreement that valence was the key driver of complexation between HA and metallic cations.

#### Cellulase activity and protein adsorption

**Fig. 5:**
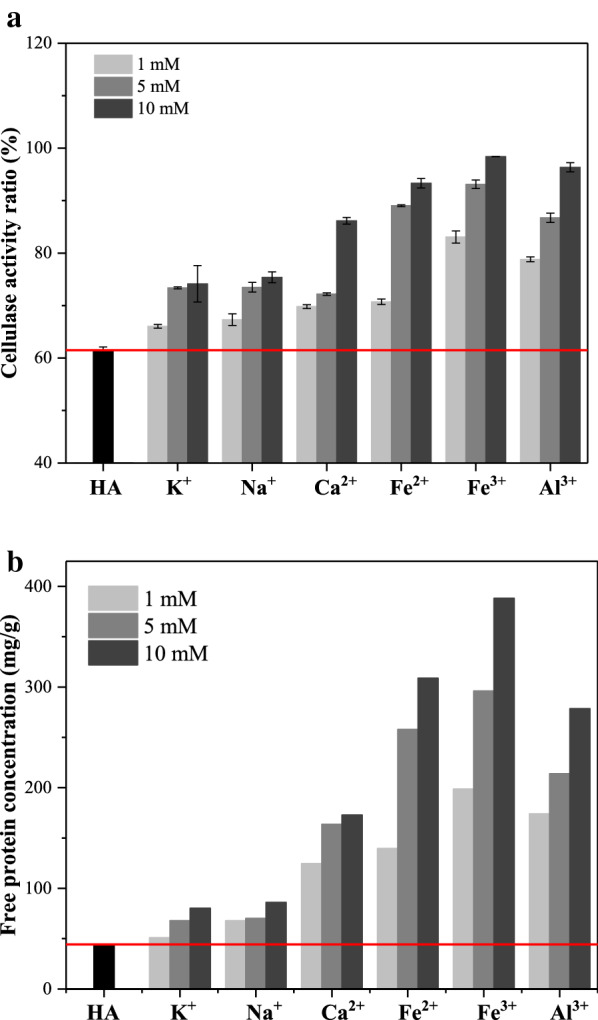
The cellulase activity ratio (**a**) and free protein concentration (**b**) of the suspension of the HA and metallic cations

With the understanding of which cations are most effective for potentially neutering HA’s negative effects on cellulase, we next endeavored to quantify how cellulase activity and protein adsorption changes as a function of cation treatment of HA. Results from Fig. 5a revealed that the cellulase activity of the treated suspensions all increased due to the complexation. The same concentration (10 mM) of K^+^, Na^+^, Ca^2+^, Fe^2+^, Fe^3+^, and Al^3+^ added to the suspension of HA resulted in the cellulase activity improving from 63.7% (control) to 74.2%, 75.4%, 86.2%, 93.3%, 98.4%, and 96.4%, respectively. Fe^3+^ again showed the greatest effect upon improving enzyme activity. In addition, we also measured the concentration of free enzyme protein in these mixed systems. These results are included in Fig. 5b. From these measurements, it can be seen that the free protein concentration of the suspension increased to varying extents due to the addition of metallic cations. This was likely because of the complex interaction of metallic cations with HA, which reduced the adsorption of cellulase by HA. The increase of cellulase activity was also likely due to the decrease in the content of enzyme protein ineffectively adsorbed by HA. Therefore, the introduction of metallic cations can reduce ineffective adsorption of HA on cellulase and in turn elevate increase cellulase activity.

### Improvement of enzymatic hydrolysis of unwashed materials with Fe^3+^

**Fig. 6:**
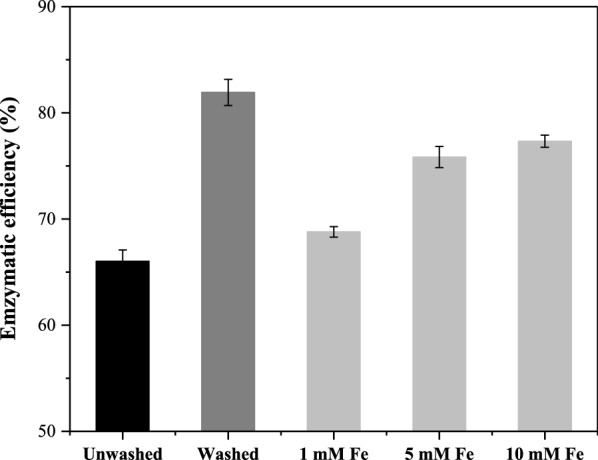
The enzymatic hydrolysis of unwashed pretreated PWWS with Fe^3+^ added

To reduce the economic costs brought by washing and increase the efficiency of enzymatic hydrolysis of HA-pretreated WWS, Fe^3+^ was applied to the enzymatic hydrolysis of AWWS as an alternative method for overcoming the negative effects of HA. The experimental data from this assay are shown in Fig. 6. It displayed that the enzymatic efficiency of the unwashed AWWS was increased to 77.3% when the 10 mM Fe^3+^ was added, while the unwashed AWWS was only 66.1%. This is likely due to Fe^3+^ complexation capacity for the residual HA in AWWS. This interaction in turn allowed for the elimination of HA’s adverse effects on cellulase, which is consistent with the previous results. The electrostatic adsorption of Fe^3+^ and HA effectively relieved the ineffective adsorption of cellulase by HA. From an economic perspective, a relatively small dosage number of metallic cations was shown to be just as effective at preserving enzymatic hydrolysis efficiency as the implementation of a large volume substrate washing step that also elevates a given biorefinery process’ environmental impact. It is our hope that the strategy demonstrated in this work will be adopted into other investigations intended to aid the realization of biorefinery processes based upon WWS.

## Conclusions

Humic acids, a natural surfactant, have been previously demonstrated as negatively influencing enzymatic hydrolysis of cellulose. It is believed that this may be due to residual humic acid from WWS pretreatment inhibiting the cellulase activity via a mechanism involving adsorption of enzyme protein. Interestingly, the addition of metallic cations (specifically Fe^3+^) resulted in observable complexation between humic acid and cations, which neutered the negative effects of humic acid upon enzymatic conversion of humic acid -pretreated WWS. Compared with other cations, Fe^3+^ is a superior additive that can effectively eliminate the effect of residual humic acid on enzymatic hydrolysis after humic acid pretreatment. This work successfully demonstrates an alternative method for decreasing the negative effects of soil-borne humic acid on biorefining of agricultural wastes.

## Methods

### Materials and reagents

Waste wheat straw (WWS) was supplied by the Shandong Quanlin Group (Shandong, China). The large amount (69.2%) of ash contained in the WWS was washed out using tap water at 1:10 (w/v) for five times, and stored at 4 °C [6]. Commercial cellulase (Cellic® CTec.2.0) was provided from Novozymes North America (Franklinton, NC). Humic acid was purchased from Sigma-Aldrich (Shanghai, China), and metallic salts (KCl, NaCl, CaCl_2_, FeCl_2_·4H_2_O, FeCl_3_·6H_2_O, AlCl_3_) was analytical grade and purchased from Nanjing Reagent Company (Nanjing, China). Bradford reagent and bovine serum protein standard solution were also purchased from Sigma-Aldrich (Shanghai, China).

### Pretreatment with HA

A dry weight of 50 g prewashed WWS was subjected to autohydrolysis with 0.1 g/g-substrate of HA in the distilled water medium. Autohydrolysis took place in 1.25 L stirred reactors (HH-SJ6CD, Youlian, China) at 180 °C for 40 min at a component mass ratio of 1:10 (w/v) WWS to the water [20]. After pretreatment, the reactors were cooled rapidly via submersion into an ice water bath. The pretreated slurry was then pressed using cheesecloth to recover the autohydrolyzed WWS (AWWS, Solid fraction). The AWWS was divided into two parts, with one half washed with deionized water thoroughly until its wash filtrate reached neutrality. The remaining half was kept unwashed. Residual HA content in unwashed AWWS was measured by forced ion exchange method under nitrogen with either a Na_4_P_2_O_7_ solution (0.5 M, pH 9.0) and a NaOH solution (0.5 M, pH 13.5) using sample-to-solvent ratios of 1:10, which is based on the method provided by IHSS (International Humus Society) [46]. Unwashed and washed AWWS were finally stored in sealed plastic bags at 4 °C.

### Enzymatic hydrolysis of unwashed and washed AWWS

Both unwashed and washed AWWS were subjected to enzymatic hydrolysis by cellulase (Cellic® CTec2) at a substrate-to-sodium citrate solution (50 mM, pH 4.8) ratio of 5% (w/v) in 150-mL bottles. When AWWS was enzymatically hydrolyzed in the presence of FeCl_3_, sodium citrate solution was made to contain 1 mM, 5 mM, and 10 mM FeCl_3_. Enzyme loadings were 20 FPU/g glucan for all experiments. Enzymatic hydrolysis was conducted at 50 °C for 72 h using a thermostat shaker (Vortex-2, Shanghai, China) at 150 rpm for slurry agitation. Individual 0.2 mL aliquots were taken at 2 h, 4 h, 6 h, 12 h, 24 h, 48 h, and 72 h. Each sample was frozen and preserved until further analysis. The collected samples were centrifuged at 10,000 rpm for 5 min to obtain a supernatant which was analyzed for sugars by HPLC (Agilent 1260 Series) [47]. Enzymatic hydrolysis efficiency of the AWWS was calculated according to the following equation:

$${\text{Enzymatic}\;\text{ hydrolysis}\;\text{efficiency}}\left( \% \right)=\frac{{{\text{Glucose}\;\text{in}\;\text{enzymatic}\;\text{hydrolysate}}\left( {\text{g}} \right) \times 0.9}}{{{\text{Glucan}\;\text{in}\;\text{AWWS}}\left( {\text{g}} \right)}} \times 100\% ,$$

where 0.9 is the factor that converts glucose to equivalent glucan. All experiments were done in duplicate.

### The complexation between HA and metallic cations

In order to investigate the interaction of HA with different metallic cations, verification experiments were conducted. First, the HA was suspended into deionized water at a concentration was 2.5 g/L (w/v). This value was based on the concentration of residual HA in unwashed AWWS. The turbid suspension was then stirred for 24 h at 25 °C to achieve homogeneity using a thermostatic oscillator (Vortex-2, Shanghai, China). System pH was measured to be 6.5 ± 0.2. After stirring time, the mixture was divided into equal parts for mixing with different species and concentrations of metallic salts (control, KCl, NaCl, CaCl_2_, FeCl_2_·4H_2_O, FeCl_3_·6H_2_O, AlCl_3_). Mixed solutions were adjusted to pH 6.5 ± 0.2 with 0.1 M NaOH. These new mixtures were again stirred for 24 h at 25 °C using the same setup described above. At the conclusion of mixing time, each suspension was centrifuged to remove the complexed HA and then stored at 4 °C for further investigation and analysis.

### Analytical methods

#### The chemical compositional analysis of raw WWS and AWWS

The chemical compositions of the raw WWS and AWWS were determined according to a protocol from the National Renewable Energy Laboratory, NREL [48]. The concentration of sugars (glucose, xylose, galactose, and arabinose) in each sample’s acid digestate was quantified by a high-performance liquid chromatography HPLC (Agilent 1260 series, Agilent Technologies, USA) using an Aminex HPX-87H column with 5 mM H_2_SO_4_ as a mobile phase at a flow rate of 0.6 mL/min at 55 °C.

#### Residual metallic cations of the complexed HA suspensions

In order to detect the number of metallic cations complexed by HA in colloidal solution, cation contents of the supernatants from HA and metallic cations mixtures were determined with an inductively coupled plasma mass spectrometer (ICP-MS, NexION300X, PerkinElmer, USA). Samples were diluted by a factor of 1000 prior to testing. The working parameters of the ICP-MS were 1.6 kW of high-frequency power, 16.0 L/min of plasma gas flow rate, 1.1 mL/min auxiliary airflow rate, 0.1 mL/min of sample lift rate. All samples were measured in triplicate and are displayed as averages.

#### Zeta potential of the complexed HA suspensions

The zeta potential of the mixture solutions was determined by an SZP meter (Mütek SZP 06) with deionized water as a blank control. Three replicates were tested for each sample and the results are presented as averages.

#### Dynamic viscosity of the complexed HA suspensions

The supernatants of metallic cations and HA suspensions were evaluated for dynamic viscosity by a rheometer (RS 6000, Haake, German) [49]. Shear rate was increased linearly from 0 to 1000 s^−1^, and the corresponding viscosity values were recorded every 10 s^−1^. Temperatures were held at 25 °C. Reported viscosity values were taken between 700–900 s^−1^.

#### Cellulase activity and free protein quantities of the HA and complexed suspensions

In order to investigate the effect of the HA and mixed suspensions on cellulase activity, the solutions complexed suspensions were mixed with 1 g cellulase (Cellic® CTec.2.0) and stirred for 24 h at room temperature. The filter paper enzyme activity and free protein content of the mixed cellulase solutions were determined.

Relative enzyme activity was assayed by adding the cellulase solution and sodium citrate buffer (pH 4.8, 50 mM) to 1 × 6 cm Whatman No. 1 filter paper (50 mg) in the test tube, and reacting the contents in a water bath at 50 °C for 60 min. The total reaction volume was 1.5 mL. Next, 3 mL DNS was added and heated above 85 °C for 10 min to cease further hydrolysis reactions. Halted solutions were then diluted to 50 mL, and solution absorbance was measured at 540 nm with a UV–visible spectrophotometer. Glucose standard solutions (1.0 g/L, 1.2 g/L, 1.4 g/L, 1.6 g/L, 1.8 g/L, 2.0 g/L) were subjected to the same operations in order to establish a glucose standard equation [30]. The filter paper enzyme activity was calculated by the following formula:

$${\text{Filter}\;\text{paper}\;\text{enzyme}\;\text{activity}}\left( {{\text{FPIU}}/{\text{mL}}} \right)= \frac{{{\text{Glucose }}\left( {\text{g}} \right)}}{180 \times 60 \times 0.5} \times {\text{Enzyme}\;\text{dilution},}$$

where the 180 g/mol is the molar mass of glucose, 60 min is the reaction time, 0.5 ml is the volume of the test enzyme solution. Each data point was averaged from two replicates.

Protein determination was using the Bradford method as reported in the literature [50]. 0.2 mL of all samples were added to 10-mL stoppered test tubes (Zhisheng, Jiangsu, China), and 3.0 mL of staining agent was then added. Meanwhile, the mixing liquids were inverted to achieve homogeneity, at which point a timer was set. After 12 min passed, the absorbance of each liquid was measured at 595 nm by a UV–visible spectrophotometer. Deionized water was used as a blank control. Bovine serum protein (0.2 mg/mL, 0.4 mg/mL, 0.6 mg/mL, 0.8 mg/mL, and 1.0 mg/mL) as a protein standard was also subjected to the same operations to create a standard curve of protein concentration. Protein concentration in all samples was calculated from the absorbance. The adsorbed protein content is based on the total added protein content minus the free protein content in the supernatant. Each data point was averaged from two replicates.

## Data Availability

All data generated and analyzed in this study are included in this published article.

## Abbreviations

WWS: Waste wheat straw
HA: Humic acid
AWWS: Autohydrolyzed waste wheat straw with 10 g/L humic acid
PEG: Polyethylene glycol
SDS: Sodium dodecyl sulfate
NREL: National Renewable Energy Laboratory
HPLC: High-performance liquid chromatography
FPA: Filter paper enzyme activity
